# Genomovar assignment of *Pseudomonas stutzeri* populations inhabiting produced oil reservoirs

**DOI:** 10.1002/mbo3.179

**Published:** 2014-06-02

**Authors:** Fan Zhang, Yue-Hui She, Ibrahim M Banat, Lu-Jun Chai, Liu-Qin Huang, Shao-Jin Yi, Zheng-Liang Wang, Hai-Liang Dong, Du-Jie Hou

**Affiliations:** 1 The Key Laboratory of Marine Reservoir Evolution and Hydrocarbon Accumulation Mechanism, Ministry of EducationChina; 2 School of Energy Resources, China University of Geosciences (Beijing)Beijing, 100083, China; 3 College of Chemistry and Environmental Engineering, Yangtze UniversityJingzhou, Hubei, 434023, China; 4 Faculty of Life and Health Sciences, University of UlsterColeraine, BT52 1SA, N. Ireland, U.K; 5 State Key Laboratory of Biogeology and Environmental Microbiology, University of Geosciences (Beijing)Beijing, 100083, China; 6 Department of Geology, Miami UniversityOxford, Ohio, 45056

**Keywords:** 16S rRNA gene, Genomovar, oil reservoirs, *Pseudomonas stutzeri*

## Abstract

Oil reservoirs are specific habitats for the survival and growth of microorganisms in general. *Pseudomonas stutzeri* which is believed to be an exogenous organism inoculated into oil reservoirs during the process of oil production was detected frequently in samples from oil reservoirs. Very little is known, however, about the distribution and genetic structure of *P. stutzeri* in the special environment of oil reservoirs. In this study, we collected 59 *P. stutzeri* 16S rRNA gene sequences that were identified in 42 samples from 25 different oil reservoirs and we isolated 11 cultured strains from two representative oil reservoirs aiming to analyze the diversity and genomovar assignment of the species in oil reservoirs. High diversity of *P. stutzeri* was observed, which was exemplified in the detection of sequences assigned to four known genomovars 1, 2, 3, 20 and eight unknown genomic groups of *P. stutzeri*. The frequent detection and predominance of strains belonging to genomovar 1 in most of the oil reservoirs under study indicated an association of genomovars of *P. stutzeri* with the oil field environments.

## Introduction

*Pseudomonas stutzeri* is a nonfluorescent widely distributed species of the genus *Pseudomonas* belonging to the gamma subclass of Proteobacteria (Bennasar et al. 1996; Lalucat et al. 2006). Strains of the species have been isolated from various environmental samples including marine sediments, soil contaminated with crude oil (Sikorski et al. 2002a, 2005; Mulet et al. 2011), clinical samples (Holmes 1986; Scotta et al. 2012) and bottle water (Papapetropoulou et al. 1994) among many others. The species has great physiological capacities including the ability to degrade environmental pollutants such as high-molecular-weight polyethylene glycols and other xenobiotics (Criddle et al. 1990; Chauhan et al. 1998; Coates et al. 1999) and the cleavage of C–N bonds in oil compounds (Kilbane et al. 2003). It also has been considered of relevance as a possible environmental reservoir of antibiotic resistance genes (García-Valdés et al. 2010) and in applications related to microbial enhanced oil recovery (EOR) (Keeler et al. 2013).

Diversity within the species is not limited to physiological properties but is also reflected at the genetic level. Previously, strains of *P. stutzeri* were classified into at least 21 genomovars (Rosselló et al. 1991; Scotta et al. 2013). The 16S rRNA gene, the internally transcribed spacer region 1 (ITS1), the genes coding for gyrase B (*gyrB*), and the D subunit of the sigma factor (*rpoD*) have been confirmed to be relevant for the phylogenetic affiliation of the species *P. stutzeri* (Bennasar et al. 1996; Sikorski et al. 2002b, 2005; Cladera et al. 2004). Additionally, fragments of the each locus could serve as excellent data sets to differentiate and establish the genetic diversity and population structure of the species of *P. stutzeri* (Yamamoto et al. 2000; Rius et al. 2001 and Cladera et al. 2004; Mulet et al. 2010, 2011).

In general, undisturbed oil reservoirs have low redox potentials and contain little oxygen and hence only strict anaerobes can be considered as truly indigenous (Magot et al. 2000). In this regard, members of the *Pseudomonas* sp. were not considered as indigenous to oil reservoirs (Orphan et al. 2000; Magot 2005) yet at least 10 studies of microbial community in samples from oil reservoirs reported its presence in these environments (Table S1). It is, therefore, important to note that due to drilling and oil recovery processes, producing oil reservoirs are dynamic environments that experience changing geochemical conditions such as the introduction of sulfate and oxygen ultimately resulting in changes to the indigenous microbial community structure (Youssef et al. 2009).

In such environments, local microorganisms may be continually introduced and those possessing exceptional survival abilities such as *P. stutzeri* can gain a lead in the formation of new ecological systems different to the original traits within surviving indigenous microbes (Youssef et al. 2009; Zhang et al. 2012). It is also well known that *P. stutzeri* possesses high physiological and genetic diversity which results from high rate genetic mutations, transpositions, and recombinations easily occurring in local natural environments (Ginard et al. 1997; Rius et al. 2001). Sikorski et al. (2002b) reported such complex composition, robust strain diversity, and directional selection in *P. stutzeri* population from marine sediment and soils while Scotta et al. (2012) demonstrated that most of the clinical strains of *P. stutzeri* belonged to genomovar 1. However, very little is known about the distribution and the genetic structure of *P. stutzeri* in the special environment of oil reservoirs.

Strains of *P. stutzeri* isolated through culture-dependent methods may provide information on the morphological, physiological, and chemical characteristics of the species in addition to the diversity of the species in the local populations. However, such information solely obtained from cultivable *P. stutzeri* strains does not provide a complete picture of the species compositional distribution in a given environment. The use of a culture-independent molecular method of construction of a clone library based on 16S rRNA gene, a widely accepted tool for molecular identification in bacterial community, therefore, became an informing supplement. 16S rRNA gene sequences of *P. stutzeri* consequently have been frequently detected in oil reservoirs without the need for culture isolation (Table1).

**Table 1:** Chemical and physical characteristics of the oil reservoirs surveyed for the presence of *Pseudomonas stutzeri* in literatures and in this study.

Location	Oil field	Oil reservoir	EOR practiced^1^		Reservoir characteristics				Samples	Relative abundance^2^ (%)	Reference
			(Year)	Water-cut	Temp (°C)	Depth (m)	pH	Cl^−^ (mg/L)			
Malaysia	Bokor	104SL	Gas lift with CO_2_ and CH_4_ (NA)	70–80%	50	733–751	7.5	13500	PW(1) FW (1)	6.38 5.26	Li et al. (2012)
		104SS	Gas lift with CO_2_ and CH_4_ (NA)	70–80%	47	619–706	7.5	13200	PW (1) FW (1)	25.26 20.68	Li et al. (2012)
Alaska	Schrader bluff	GMR75	Water-flooded	NA	27	1000	7.4–7.7	6141	PW(1)	NA	Pham et al. (2009)
Brazil	Potiguar	NA	NA	80–90%	42.2	535.5–540.5	NA	30000	PW (1)	0.76	Silva et al. (2013)
China	Shengli	Gudao	Water-flooded (1974)	95%	69	1173–1230	7.2–7.5	3138	PW (1)	66.7	Ren et al. (2011)
China	Shengli	Menggulin	Water-flooded (1989)	95%	37	806	8.2–8.6	NA	PW (1)	6.7	Tang et al. (2012)
China	Shengli	Baologe	Water-flooded (2001)	78%	58.4	1380	8.4–9.2	NA	PW (2)	55.5, 82.6	Tang et al. (2012)
China	Huabei	Block M	Water-flooded (NA)	NA	37	NA	6.7	502.5	PW (1)	8.8	Zhao et al. (2012)
China	Huabei	Block B	Water-flooded (NA)	NA	58.4	NA	7.2	507.8	PW (1)	8.1	Zhao et al. (2012)
China	Kalamay	Block Q	Water-flooded (1974)	NA	32	NA	7.6	4550	PW (1)	3.7	Zhao et al. (2012)
China	NA	Qinghuang	Water-flooded (2003)	NA	65	1100–1300	7.1–7.6	NA	PW (1)	NA (<1)	Li et al. (2007)
China	Xinjiang	No. 6	Water-flooded (1973)	69.5%	25	800	7.5	3012	IW (1)	3	Zhang et al. (2012)
China	Dagang	Kong 2	Water-flooded (1975)	94.9%	55	1400	7.2	4617	PW(1) IW (1)	50 0.9	Zhang et al. (2012)
China	Henan	V4	Water-flooded (1977)	95%	70	1355	7.3	7995	PW(1) IW (1)	3.7 0.8	Zhang et al. (2012)
Middle east	Arabian	NA	NA	NA	NA	NA	NA	NA	Oil (1)	5.3	Yamane et al. (2008)
Japan	Minami-Aga	NA	NA	NA	NA	NA	NA	NA	Oil (1)	5.2	Yamane et al. (2008)
Japan	Sagara	NA	NA	NA	NA	>600	NA	NA	Core (7)	100, 22.2, 100, 4.3, 6.3, 7.7, 26.3	Nunoura et al. (2006)
China	Qinghai	Gasi	Water-flooded (NA)	70%	55	2200	7.6	91425	PW (1)	3.7	This study
China	Jianghan	Wangxie	Water-flooded (1973)	86%	80	1500−1800	7.9	150513	PW (1)	3.1	This study
China	Tuha	Qiuling	Water-flooded (1995)	87%	35	2600–2800	7.5	5346	IW (2)	62.8, 34.4	This study
China	Dagang	Yangerzhuang	Water-flooded (1975)	95%	55	1800	7.2	4761	PW (2)	31, 12.2	This study
China		Yangcong	Water-flooded (1975)	94.1%	50	2000	7.3	5024	PW(2)	60.5, 61.2	This study
China		Kongdian	Water-flooded	93%	50–60	1300–1400	7.2	5362	PW (2)	48.2, 85.6	This study
China	Shengli	Gudao	Water-flooded (1983) Polymer-flooded (1993)	96%	70	1100–1240	7.6	3875	PW (1)	71.1	This study
China	Daqing	N2	Water-flooded (1978) Polymer-flooded (1995–2003)	97%	40–45	1300	7.9	525	PW (3)	64.4, 50, 39.3	This study

NA, Characteristics of petroleum reservoirs surveyed in this study were collected from literature or supplied by oilfield operators. In many cases the information was incomplete. Where data could not be obtained an NA has been recorded; PW, samples collected from production wells; FW, samples formation water; IW, samples from injection wells.

1 EOR presents methods of enhanced oil recovery.

2 Relative abundance presents the relative abundance of *P. stutzeri* in each clone library constructed from each sample collected.

The main aim of this study was to determine the distribution and the genomovar assignment for *P. stutzeri* in the oil reservoirs environment by grouping 16S rRNA gene sequences of *P. stutzeri*, obtained using both culture-dependent and culture-independent methods.

## Materials and Methods

### Reference sequences collection

References that reported microbial communities of oil reservoirs were screened and oil reservoirs in which clones of *P. stutzeri* were reported were selected. 16S rRNA gene sequences of the different *P. stutzeri* clones were retrieved from the GenBank database of the National Center for Biotechnology Information (NCBI) (http://www.ncbi.nlm.gov) using the accession numbers reported in the references. The main characteristics of these reservoirs are listed in Table1.

Another part of 16S rRNA gene reference sequences was obtained from strains which have been grouped into genomovars 1–22 of *P. stutzeri* and were also retrieved from NCBI using the accession numbers reported in literatures (Bennasar et al. 1996; Sikorski et al. 2002a; Sikorski et al. 2005 Cladera et al. 2004).

### Sampling from oil reservoirs

Mixed oil/water liquids were retrieved directly from wellheads of production wells and injection wells and stored in sterile plastic bottles filled completely and transported to the laboratory for cultural and molecular analyses. Numerous samples were collected from different oil fields including those subjected to water and polymer flooding. As we focused on the species of *P. stutzeri*, only samples that contained *P. stutzeri* were used in this study. Characteristics of oil reservoirs in which sequences of *P. stutzeri* were detected are shown in Table1.

### 16S rRNA clone library construction and taxonomic classification and phylogenetic analysis

All samples from oil reservoirs were subjected to DNA extraction following the manufacturer's protocol for the FastDNA Spin Kit for Soil (Qbiogen, Carlsbad, CA) as previously described (Zhang et al. 2010, 2012). The bacterial 16S rRNA gene in the bulk DNA was amplified with the universal bacteria-specific primers of 27F and 1492R (Zhang et al. 2010). The PCR reacting system (25 *μ*L) contains 2.5 *μ*L of 10 × PCR buffer (Mg^2+^ plus), 10 nmol of deoxynucleotide triphosphates, 10 pmol of each primer, 1 U Taq DNA polymerase (TakaRa, Dalian, China), and 1 *μ*L of template DNA. The thermal cycling conditions were as follows: an initial denaturation at 94°C for 5 min, 40 cycles of 94°C for 30 sec, 56°C for 60 sec, 72°C for 90 sec, and a final extension step of 72°C for 10 min. Amplified fragments were ∼1450 bp.

After being purified with an Agarose Gel DNA Purification Kit (TianGen Biotech, Beijing, China), amplicons of 16S rRNA genes were cloned into Trans1-T1 competent cells (TransGen Biotech, Beijing, China) using PGEM-T Easy Vector (Promega, Madison, WI). Overall, 150 putative clones (white) from each plate were chosen randomly. A reamplification, with sets of vector-specific primers T7/SP6, was taken to determine positive clones. PCR products of positive clones were clustered into different operational taxonomic units (OTUs) using amplified ribosomal DNA restriction analysis (ARDRA) with *Hinf*I and *Hae*III (TaKaRa). An ABI PRISM 3730 DNA sequencer (SinoGenoMax Co., Ltd., Beijing, China) was used to gain sequences of the representative clones.

All detected sequences in clone libraries were manually trimmed and edited using DNAMAN version 5.2.2.0. The obtained sequences were submitted to NCBI for Basic Local Alignment Search Tool (BLAST) algorithm of nucleotide to determine their phylogenetic affiliations.

### Isolation and identification of *Pseudomonas stutzeri* from samples of oil reservoirs

*Pseudomonas stutzeri* strains were isolated from environmental samples of oil reservoirs, using a culture enrichment procedure in two liquid media SW-DAN (seawater medium for aerobic denitrifiers with ammonia as an additional source of nitrogen) and SW-DNN (seawater medium for aerobic denitrifiers with nitrate as the sole nitrogen source). SW-DAN medium supplemented with, per liter, 0.5 g (NH_4_)_2_SO_4_, 5.95 g sodium succinate and SW-DNN supplemented with, per liter, 2 g KNO_3_, 18.7 g sodium succinate (Mulet et al. 2011) in an artificial seawater medium (SW) which contained per liter, 24 g NaCl, 10.5 g MgSO_4_·7H_2_O, and 0.11 g NaHCO_3_ (Sikorski et al. 2002b). The pH was adjusted to 7.5–8.0, and for SW-DAN and SW-DNN plate cultures 20 g agar was added.

Five milliliter of original mixed oil/water liquid was inoculated into 30 mL of liquid SW-DAN and SW-DNN medium and incubated aerobically at 30°C on an orbital shaker at 150 rmp/min for 4 days. After three successive subculturing on both media, 200 *μ*L of culture at 10^−3^, 10^−4^ and 10^−5^ obtained by 10-fold dilution method was plated on the same solid media and incubated at 30°C for 4 days. Colonies with different morphology were streaked twice on SW-DAN and SW-DNN for single colony isolation. Cells of the purified isolates were inoculated into 5 mL of liquid SW-DAN and SW-DNN and incubated at 30°C at 150 rmp/min overnight. All cultures obtained were tested for identification of *P. stutzeri*.

A quantity of 1 mL culture of purified isolate was centrifuged at 10000*g* for 8 min for cell collection. DNA in the pellet cells was extracted following the manufacturer's protocol for the DNA Extraction Kit (TianGen Biotech). The DNA obtained was detected using agarose gel electrophoresis. Amplification of 16S rRNA gene from genomic DNA was carried out as described previously (Zhang et al. 2010). Amplified fragments were purified with agarose Gel DNA Purification Kit (TianGen Biotech) and were sequenced and identified as described above. All sequences affiliated with *P. stutzeri* (99%, identity) were identified and used in analysis of genomovar distribution.

### Phylogenetic analysis

**Figure 1:**
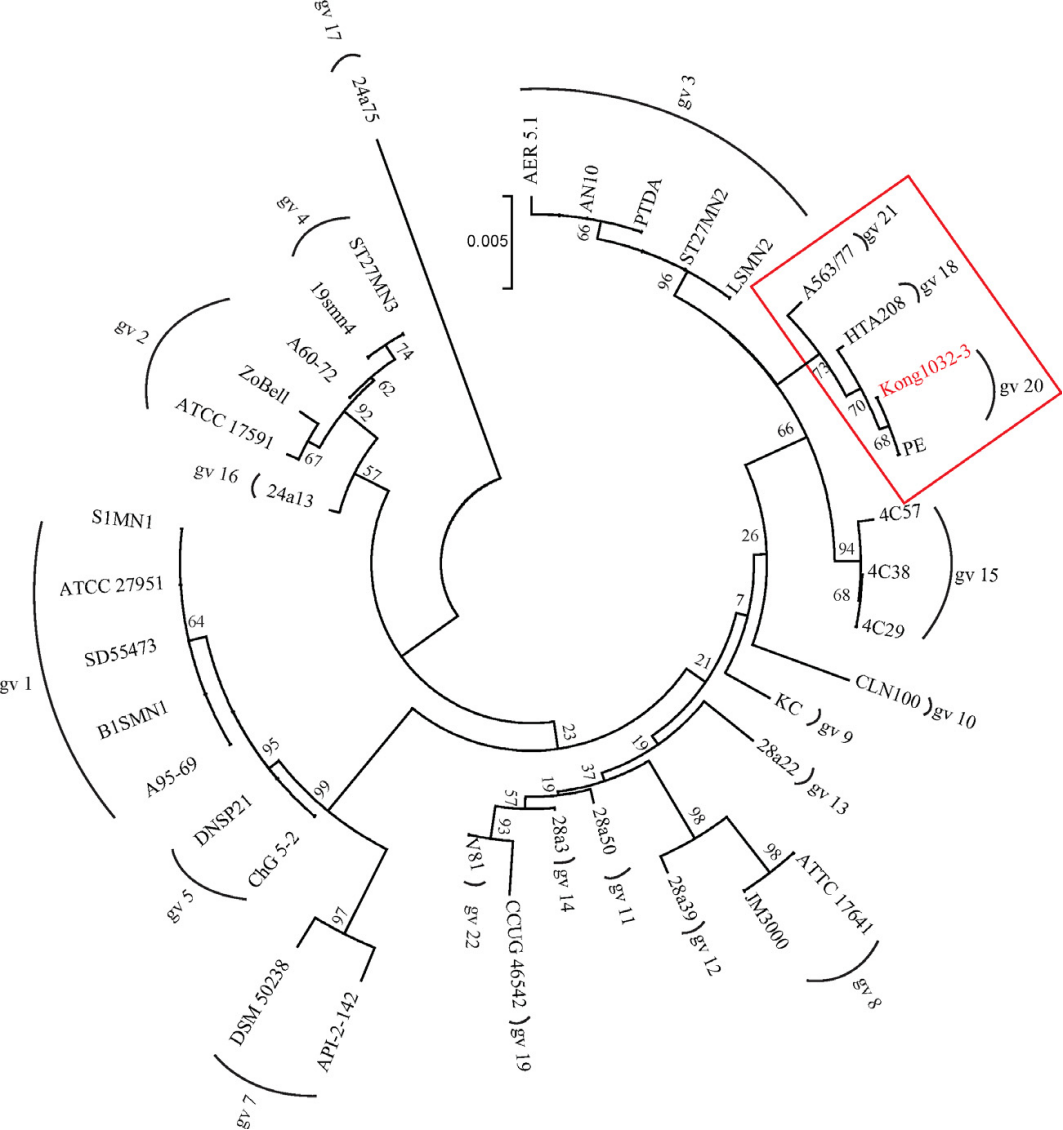
Phylogenetic tree of all 37 *Pseudomonas stutzeri* strains representing the established genomovars 1–22 based on the analysis of 16S rRNA gene. The evolutionary distances were computed using the Maximum Composite Likelihood method. The bootstrap test (1000 replicates) are shown next to the branches. Dendrograms were generated using neighbor-joining method. Clone of K1032-3 is clustered to gv 20 clearly.

The retrieved 16S rRNA gene sequences of genomovars 1–22 were aligned using computer program of the Clustal W with a number of bootstraps of 1000 in the software of the BioEdit (version7.0.9). Dendrograms were generated by neighbor-joining method included in the software of the MEGA 5. Phylogenetic tree based on these 16S rRNA gene sequences of the established *P. stutzeri* genomovars of 1–22 is displayed in Figure1.

In order to establish the genetic diversity and population structure of *P. stutzeri* detected in samples from oil reservoirs, the 16S rRNA gene sequences of *P. stutzeri* cited in literature, detected in clone libraries and obtained from isolates in this study were aligned together. The alignment and construction of phylogenetic tree followed the steps above.

On the basis of the 16S rRNA gene sequences retrieved from reported literatures, 37 reference strains representing the established genomovars 1–22 were integrated into a dendrogram (Fig.1). In this dendrogram, notably, the former genomovar 6 was excluded as it has been reclassified to as a species of *Pseudomonas balearica* (Bennasar et al. 1996). The sequence diversity characteristic of the species displayed in the dendrogram confirmed previous studies that phylogenetic tree based on 16S rRNA gene sequences correlated well with the subgroups termed genomovars (Bennasar et al. 1996; Sikorski et al. 2002a, 2005; Cladera et al. 2004; Scotta et al. 2013). These reference strains were isolated mainly from marine, clinical, and soil environment and not a single strain was reported from the environment associated with oil reservoirs (Table S1).

### Nucleotide sequence accession number

The nucleotide sequences detected in this study were submitted to the GenBank database under the following accession numbers: KC796755–KC796783 (detected sequences of *P. stutzeri* in this study) and KC796784–KC796794 (isolates of *P. stutzeri*). The accession numbers of the 16Sr RNA gene sequences of the reference strains are listed in Table S1.

## Results

### *Pseudomonas stutzeri* detected in produced oil reservoirs

We obtained 29 *P. stutzeri* sequences, detected in 28 samples from 17 oil reservoirs by researchers worldwide through their 16S rRNA gene clone libraries, and used as reference sequences in this study. Additionally, in our laboratory 30 sequences of *P. stutzeri* were detected in 14 samples from eight oil blocks. These sequences affiliated to *P. stutzeri* were reported/detected in samples collected from production wellheads, injection wells, crude oils, and cores of oil reservoirs in Malaysia (Li et al. 2012), Alaska (Pham et al. 2009), Brazil (Silva et al. 2013), Middle East (Yamane et al. 2008), Japan (Nunoura et al. 2006; Yamane et al. 2008) and China (Li et al. 2007; Ren et al. 2011; Tang et al.^2012^; Zhang et al. 2012; Zhao et al. 2012). The main characteristics of these reservoirs and the enhanced oil recovery process (EOR) practiced are listed in Table1. These reservoirs had an in situ temperature range of 25–80°C, depth range of 535.5–2800 m, pH of produced fluids range of 6.7–9.2, and a Cl^−^ range concentration of 502–150513 mg/L. Most of these oil reservoirs have been subjected to water-flooding for decades to enhance oil recovery with the fraction of water being co-produced with oil ranging from 70% to 97%. Exceptionally, the oil reservoir in Bokor oil field in Malaysia was subjected to gas lift with CO_2_ and CH_4_, and Gudao and N2 oil reservoirs in Shengli and Daqing oil fields, respectively, had undergone polymer flooding with polyscrylamide. The relative abundance of *P. stutzeri*-related sequences from the total 43 clone libraries reported in literature and constructed within this study varied from 0.76% to 100% (Table1).

### Compositional distribution and genomovar assignment of *Pseudomonas stutzeri* in oil reservoirs

**Figure 2:**
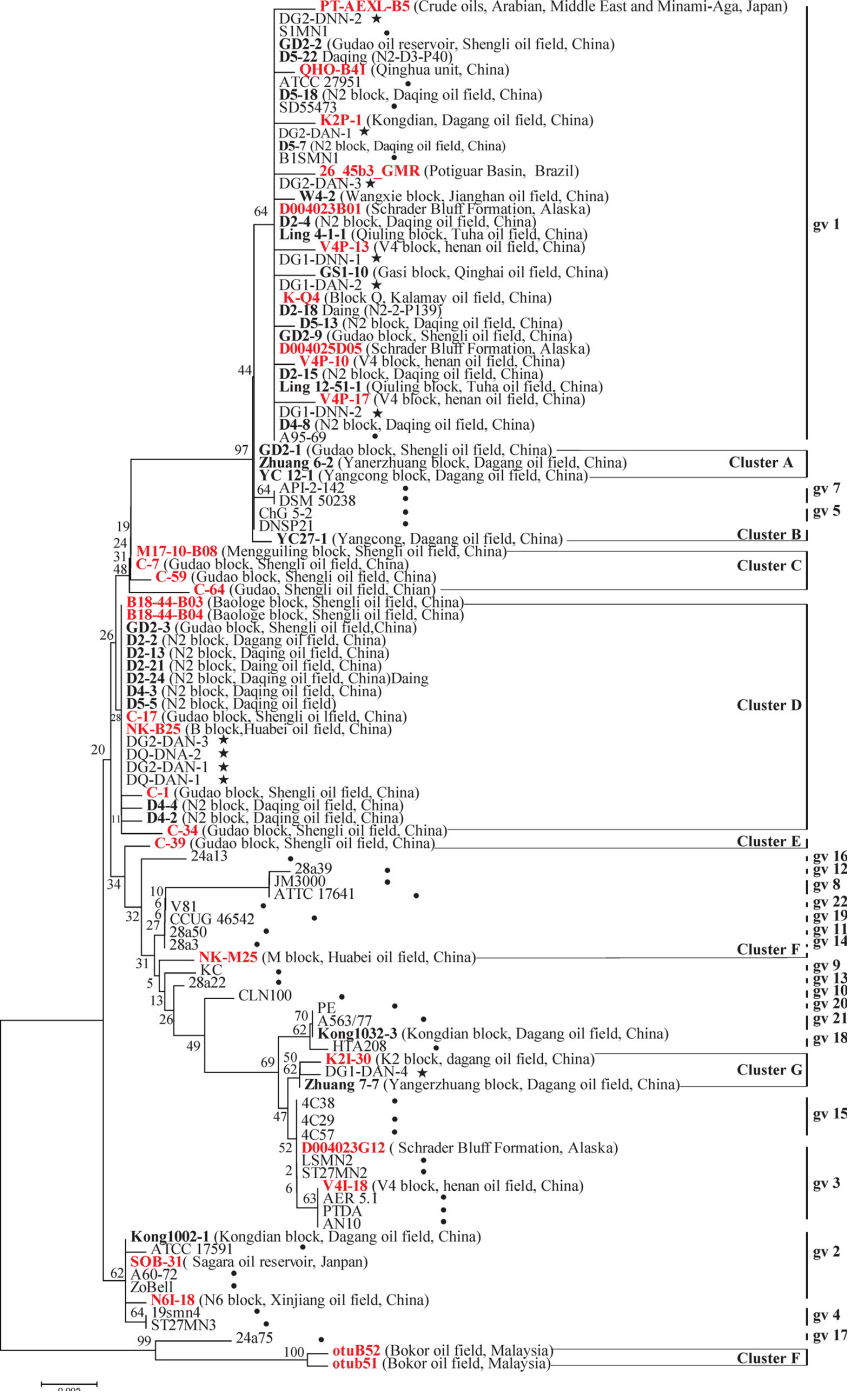
Phylogenetic tree of all 107 *Pseudomonas stutzeri* strains based on the analysis of 16S rRNA gene. The evolutionary distances were computed using the Maximum Composite Likelihood method. The bootstrap test (1000 replicates) are shown next to the branches. Dendrograms were generated by neighbor-joining method. Location information of all clones are included. Sequences clustered into A–H groups are proposed as new genomovars. The clones colored red were *P. stutzeri* detected in oil reservoirs from literatures, the clones in bold black were new *P. stutzeri* detected in this study, and the strains recorded with ✶ are new *P. stutzeri* isolated in this study, while the strains recorded with • are *P. stutzeri* represents the established genomovars 1–22.

**Figure 3:**
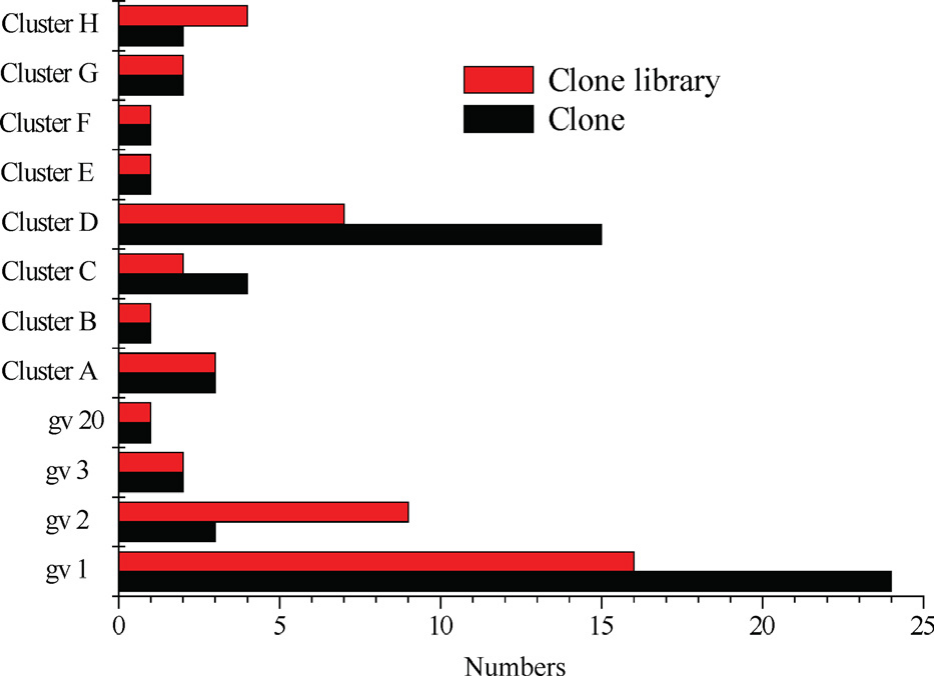
Results from 59 *Pseudomonas stutzeri* clones detected in 42 clone libraries constructed from oil reservoir samples were evaluated for the genomovar assignment of *P. stutzeri* in number. The bars colored red represent the numbers of the clone library in which *P. stutzeri* was detected, and the bars in black represent the numbers of *P. stutzeri* clone. Overall *P. stutzeri* affiliated with genomovar 1 (24 clones from 16 clone libraries) is the most frequently detected group in the oil field environments.

Phylogenetic relationships of the 16S rRNA gene sequences of *P. stutzeri* collected in the clone libraries reported in literature and detected in this study and belonging to genomovars 1–22 are shown in Figure2. On the basis of the dendrogram, twenty-four 16S rRNA gene sequences of *P. stutzeri* (10 from references and 14 from this study) were grouped into genomovar 1. These 24 sequences were from 16 samples (Fig.3); eight from literature and eight from this study obtained from production wells collected in oil reservoirs of Shengli, Daqing, Tuha, Qinghai, Qinghuang, Kalamay, Dagang, Henan, Potiguar, and Schrader bluff oil fields, and two samples of crude oils collected in Middle East and Japan. Two 16S rRNA gene sequences of D004023G12 and V4I-18 detected in samples from oil reservoirs of Henan and Schrader bluff oil field, respectively, were affiliated closely with genomovar 3. Three Sequences of clones of SOB-31, N6I-18, and K1002-1 detected in core samples collected in Sagara oil reservoir, an injection well in Xingjiang oil field and a production well in Dagang oil field, respectively, belonged to genomovar 2. Sequence of K1032-3 could not be identified in Figure2 but showed closer relationship to the strains PE (gv 20) in Figure1.

Twenty-nine detected 16S rRNA gene sequences of *P. stutzeri* (15 from references and 14 from this study) were grouped outside the established genomovars and were assigned into eight clusters (A−H) within this study (Fig.2). Cluster A included two sequences detected in samples from Dagang and one sequence from Shengli oil field. Cluster B, E, and F each had only one representative sequence from Dagang, Shengli, and Huabei oil field, respectively. Cluster C was composed of four sequences detected in samples from in Shengli oil field. Cluster D seemed to be the largest and included 15 detected sequences from Shengli, Daqing, and Hubei oil field. Cluster G contained only sequences from Dagang oil reservoir, and cluster H contained only sequences from Bokor oil field (Fig.3).

Additionally, in this study, three mixed oil/water liquids in which *P. stutzeri* was detected were subjected to the isolation procedure for *P. stutzer*i in SW-DAN and SW-DNN. Two liquids were from water-flooded wells of Zhuang 7 (DG1) and K1002 (DG2) in Dagang oil field and one liquid was from a polymer-flooded well of D2 (DQ) in Daqing oil field. A total of 17 isolates with different color and morphology were obtained. Fifteen strains (88.2% of the total isolates) were identified as *P. stutzeri* based on algorithm of 16S rRNA gene sequence by the BLAST in GenBank database of NCBI. Twelve strains were isolated from Dagang oil field and five from Daqing oil field. Not a single strain of *P. stutzeri* was isolated from the sample collected from Daqing oil field when the medium of SW-DNN was used. Two strains isolated from Daqing oil field using the medium of SW-DAN were not affiliated with *P. stutzeri* but were *Acinetobacter* sp.

On the basis of the 16S rRNA gene sequences, 11 representative isolates were also integrated into the dendrogram of all 16S rRNA gene sequences used in this study (Fig. S1). Six strains of DG2DNN-2, DG2DNN -1, DG1DNN -1, DG1DNN -2, DG1DAN-2, and DG1DAN-3 belong to genomovar 1. Four strains of DG2DAN-1, DG2DAN-3, DQDAN-1, and DQDAN-2 were grouped in cluster D and one strain of DG1DAN-4 was grouped in cluster G both of which not assigned to any known genomovars. The distribution and the genomovar assignment of *P. stutzeri* isolated in oil reservoirs by culture-dependent method were roughly consistent with those of the genus detected by culture-independent method.

It is important to note that in this study strains grouped into genomovar 1 contained isolates using both media for denitrifiers; however, strains grouped into cluster D were all isolated in SW-DAN medium for denitrifiers metabolizing with ammonia nitrogen, which was consistent with that *P. stutzeri* grouped into cluster D were mainly from oil reservoirs undergone polymer-flooding with polyacrylamide which can produce ammonium through hydrolysis.

## Discussion

*Pseudomonas stutzeri* habited a wide range of oil reservoirs with different characteristics. Oil recovery processes seemed to be the main factor for its appearance in oil reservoirs. Two studies of phylogenetic diversity of microbial community in the non-flooded oil reservoirs in Niibori oil field in Japan (Kobayashi et al. 2012) and the Troll C platform (Dahle et al. 2008) showed no single clone of *P. stutzeri* detected. It is important to note that oil recovery processes could not be considered as an absolute indicator for the existence of *P. stutzeri* in oil reservoirs. Other complex, correlative, and invisible characteristics of the producing oil reservoirs need to be taken into consideration, which may account for the many perplexing appearances of phylogenetic diversity of microbial communities. For example, *P. stutzeri* had high abundances in some samples but was not detected in others which were collected from oil wells in the same oil reservoir after undergoing water-flooding (Ren et al. 2011; Tang et al. 2012).

This is the first study on the compositional distribution and the genomovar assignment of *P. stutzeri* in the environments of oil reservoirs. On the basis of 16S rRNA gene sequence analysis of numerous *P. stutzeri* detected in the oil reservoirs under this study, *P. stutzeri* inhabited in these oil reservoirs included sequences affiliated with four known genomovar 1, 2, 3, and 20 and eight genomic groups that may represent new genomovars which require further taxonomic studies involving DNA–DNA hybridization, sequencing of internally transcribed 16S–23S rRNA gene spacer region (ITS1), and basic physiological properties (Sikorski et al. 2005). There were complex diversity associated with *P. stutzeri* detected in oil reservoirs and those belonging to genomovar 1 were detected in almost all of the oil reservoirs. The results obtained show an association of genomovars of *P. stutzeri* with the particular geographical environment of oil reservoirs as the genomovar three members preferentially exist in marine and the genomovar seven members mainly in soil habitats contaminated by petrochemicals or other pollutions (Sikorski et al. 2002a).

While interpreting the existence of complex diversity of *P. stutzeri* in the environment of oil reservoirs, we should take into account two main factors. The first one is the origination of *P. stutzeri* strains in oil reservoirs. Notably, it was suggested that they are exogenous organisms which were introduced to oil reservoirs during drilling and oil recovery procedures and survived gradually (Magot et al. 2000; Orphan et al. 2000; Youssef et al. 2009). Therefore, their origin may be derived from inoculation of oil reservoirs with surface *P. stutzeri* within the injecting water and recycled water after being exposed to surface conditions. Cells of some genomovars (genomovar 1 in this study) might be physiologically flexible which allows them to successfully occupy the habitat of oil reservoirs and survive (Sikorski et al. 2002a). Alternately the second factor may be based on the special stresses in oil reservoirs including abiotic factors or community-related conditions which increase mutation frequencies (Finkel and Kolter 1999; Radman 1999) and transposition speed (Oliver et al. 2000) of *P. stutzeri*, which may enhance the selection chance of mutant strains (Papadopoulos et al. 1999). These kinds of gene variation mechanisms may frequently occur in *P. stutzeri* which is a species with high rearranged chromosomes and no long-range conservation of genetic map (Ginard et al. 1997). Moreover, *P. stutzeri* is capable of natural transformation resulting in genomic diversification by recombination (Carlson et al. 1983; Sikorski et al. 2002a,b).

Our results indicate that *P. stutzeri* belonging to genomovar 1 predominates appearing most frequently in oil reservoirs with different geographical locations (Table1). Although strains of genomovar 1 were isolated from soil as well as aquatic habits, their frequent appearance in oil reservoirs attracted our attention to the common geological characteristics of oil reservoirs. When strains of *P. stutzri* are introduced into oil reservoirs with injected fluids, the physico-chemical characteristics of oil reservoirs, such as temperature, pH, and electron donors and acceptors would surely have an effect on their survival, abundance, and diversity. In addition to the likelihood of originating from injected liquids, common geological characteristics of oil reservoirs must be taken into account to interpret the frequent detection of *P. stutzeri* strains belonging to genomovar 1.

Oil reservoirs are mainly subsurface environments with low redox potentials due to isolation from surface water (Magot et al. 2000). This means the available electron donors can be limited to hydrogen, volatile fatty acids (Fisher 1987), petroleum hydrocarbons, and inorganic electron donors (e.g., sulfide) while electron acceptors minimally include sulfate, carbonate, and iron (III), moreover, nitrate and oxygen are limiting in most oil reservoirs unless added with injected fluids (Youssef et al. 2009). It appears members belonging to *P. stutzeri* genomovar 1 may possess a greater ability to acclimatize to the special niches of oil reservoirs or have higher advantages to be able to achieve such capacity than other genomovars of *P. stutzeri*.

Members of different genomovars of *P. stutzeri* from the similar geographical environment belong to different ecological subpopulations inhabiting own ecological niches and constitute different evolving units (Palys et al. 1997). Our results showed sequences of *P. stutzeri* only in respective oil reservoirs which could be considered to be evolving units in own ecological niches. Cluster H (Fig.1) included two sequences from Bokor oil field which is an oil reservoir not being subjected to water-flooding but gas lift with CO_2_ and CH_4_. Cluster G comprising sequences only from Dagang oil field. Notably, cluster C and D mainly consisted of sequences from oil reservoirs subjected to polymer flooding with polyacrylamide. Cluster B, E, and F, each, only contained one sequence. All these representative sequences seemed to associate with some unusual characteristics in these producing oil fields. These unusual characteristics, therefore, may act as the special stress factors for the evolution of *P. stutzeri* in the natural habitats, resulting in the genomic diversity of the species in the environment of oil reservoirs.

In this study, strains of *P. stutzeri* isolated in our laboratory were grouped into genomovar 1, cluster D and G, which seemed roughly to be consistent with the distribution of *P. stutzeri* in the oil fields of Dagang and Daqing. Notably, not a single strain of *P. stutzeri* was isolated from the sample of D2 collected from Daqing oil field where *P. stutzeri* of genomovar 1 was noticed to be dominant in the clone library constructed from the sample of D2. This demonstrated the belief that compositional distribution of microorganisms in natural habits could not be solely established based on culture-dependent methods as it does not take into account the discrepancies in growth environments between nature and laboratories (Ward et al. 1990; Singleton et al. 2001) leading to detectable microorganisms not yet cultured. Although culture-dependent methods are not sufficient to obtain a full picture of microbial ecology, further investigation of microbial metabolism activity and determination of the genomovars of the unassigned groups must depend on these isolates.

Our results were based on the analysis of 16S rRNA gene sequences of *P. stutzeri* in 16S rRNA gene clone libraries. Although 16S rRNA gene sequences of *P. stutzeri* do not provide high resolution of all genomovars as would using special genes of *P. stutzeri* such as the *rpoD* (Cladera et al. 2004; Mulet et al. 2011; Scotta et al. 2012, 2013), our results show that using of 16S rRNA gene sequences seemed to be applicable for the purpose due to the following reasons: (1) with the studies in microbial ecology of oil reservoirs, more 16S rRNA gene data of microbial community detected in oil reservoirs were reported, which provided a good resource for us to collected information worldwide. (2) 16S rRNA gene clone library, showing a clear picture of a microbial community of bacteria (Palys et al. 1997), can provide information concerning the relative abundance of different bacterial groups. In this study, *P. stutzeri* was dominant in some samples and were slight in others, which easily demonstrated relative abundance perception of *P. stutzeri* in oil reservoirs. (3) 16S rRNA gene sequences used in the study were obtained by culture-independent methods, which reflect the microbial community more realistically than would culture-dependent methods due to uncultivable characteristic of microbes in laboratories.

## Supporting Information

Table S1 Information of 107 sequences of strains or clones of *Pseudomonas stutzeri* that have been grouped into established genomovars 1**–**22 (37), were detected in samples from oil reservoirs (59) and were isolated from oil reservoirs (11) used this study.
