# Supplementary material for: Genomovar assignment of Pseudomonas stutzeri populations inhabiting produced oil reservoirs
Source: Microbiologyopen. 2014 Jun 2;3(4):446–56. doi: 10.1002/mbo3.179 (PMC4287174; doi:10.1002/mbo3.179)
Supplement: Table S1 — Information of 107 sequences of strains or clones of Pseudomonas stutzeri that have been grouped into established genomovars 1–22 (37), were detected in samples from oil reservoirs (59) and were isolated from oil reservoirs (11) used this study. [file mbo30003-0446-sd1.doc]

**Supplementary Table 1.** Information of 107 sequences of strains or clones of *Pseudomonas stutzeri* that have been grouped into established genomovars 1**–**22 (37), were detected in samples from oil reservoirs (59) and were isolated from oil reservoirs (11) used this study.

| ***P. stutzeri* established 1– 22 genomovar** | | | | |
| --- | --- | --- | --- | --- |
| **Name** | **Gv** | **Accession number** | **Origin** | **Reference** |
| S1MN1 | 1 | AJ633557 | Wastewater | Lalucat et al., 2006 |
| ATCC27951 | 1 | AJ633553 | Yogurt | Lalucat et al., 2006 |
| SD55473 | 1 | AJ633554 | Clinical | Cladera et al., 2004 |
| B1SMN1 | 1 | AJ633556 | Wastewater | Lalucat et al., 2006 |
| A95-69 | 1 | AJ633555 | Clinical | Holmes, 1986 |
| A60-72 | 2 | AJ633560 | Clinical | Holmes, 1986 |
| ZoBell | 2 | U26420 | Marine | Bennasar et al., 1996 |
| ATCC17591 | 2 | U26261 | Clinical | Bennasar et al., 1996 |
| ST27MN2 | 3 | U25280 | Marine | Lalucat et al., 2006 |
| LSMN2 | 3 | AJ633559 | Marine | Lalucat et al., 2006 |
| PTDA | 3 | AJ633562 | Putidoil bioremediation | Cladera et al., 2004 |
| AER 5.1 | 3 | AJ633561 | Aircraft oil contaminated soil | Lalucat et al., 2006 |
| AN10 | 3 | U22427 | Marine | Lalucat et al., 2006 |
| ST27MN3 | 4 | U26419 | Marine | Lalucat et al., 2006 |
| 19SMN4 | 4 | U22426 | Marine | Lalucat et al., 2006 |
| DNSP21 | 5 | U26414 | Wastewater | Lalucat et al., 2006 |
| ChG5-2 | 5 | AF054935 | Marine | Sikorski et al., 2002 |
| DSM 50238 | 7 | U26416 | Soil | Lalucat et al., 2006 |
| API-2-142 | 7 | AJ41087 | Wastewater | Sikorski et al., 2002 |
| JM300 | 8 | X98607 | Soil | Rosselló-Mora et al. 1996 |
| ATTC17641 | 8 | AJ006106 | Clinical | Sikorski et al., 2002 |
| KC | 9 | AF067960 | Aquifer | Sepúlveda-Torres et al., 2001 |
| CLN100 | 10 | AJ544240 | Deposit of chemical products | Lalucat et al., 2006 |
| 28a50 | 11 | AJ312162 | Soil | Sikorski et al., 2005 |
| 28a39 | 12 | AJ312161 | Soil | Sikorski et al., 2005 |
| 28a22 | 13 | AJ312167 | Soil | Sikorski et al., 2005 |
| 28a3 | 14 | AJ312163 | soil | Sikorski et al., 2005 |
| 4C57 | 15 | AJ270454 | Marine sediment | Sikorski et al., 2005 |
| 4C29 | 15 | AJ270456 | Marine sediment | Sikorski et al., 2005 |
| 4C38 | 15 | AJ270457 | Marine sediment | Sikorski et al., 2005 |
| 24a13 | 16 | AJ270451 | Soil contaminated with mineral oil | Sikorski et al., 2005 |
| 24a75 | 17 | AJ312229 | Soil contaminated with mineral oil | Sikorski et al., 2005 |
| HTA208 | 18 | AB002660 | Marine | Sikorski et al., 2002 |
| CCUG 46542 | 19 | AB176955 | marine ascidian | Mulet et al., 2008 |
| PE | 20 | HF571101 | Putidoil seeded bioreactor | Mulet et al., 2011 |
| A563/77 | 21 | HF571102 | Clinical | Holmes, 1986 |
| V81 | 22 | FN99527 | Oil contaminated sand | Mulet et al., 2011 |
| ***P. stutzeri* detected in oil reservoirs** | | | | |
| PT-AEXL-B5 | 1 | AB369005 | Arabian, Middle East | Yamane et al. (2008) |
|  | 1 | AB369005 | Minami-Aga, Japan | Yamane et al. (2008) |
| GD2-2 | 1 | KC796755 | Gudao, Shengli, China | This study |
| W4-12 | 1 | KC796776 | Wangxie, Jianghan, China | This study |
| QHO-B41 | 1 | DQ675028 | Qinghuang, China | Li et al. (2001) |
| D5-18 | 1 | KC796775 | N2 blcok, Daqing, China | This study |
| K2P-1 | 1 | AY770933 | Kongdian, Dagang, China | Zhang et al. (2013) |
| D5-7 | 1 | KC796773 | N2 block, Daqing, China | This study |
| 45b3 | 1 | JN613851 | Potiguar Basin, Brazil | Silva et al. (2012) |
| K1002-5 | 1 | KC796758 | Kongdian, Dagang, China | This study |
| D004025D05 | 1 | EU7121823 | Schrader bluff, Alaska, USA | Pham et al. (2009) |
| D2-4 | 1 | KC796761 | N2 block, Daqing, China | This study |
| Ling4-1-1 | 1 | KC796757 | Qiuling,Tuha,China | This study |
| V4P-13 | 1 | JQ51877 | V4 block, Henan, China | Zhang et al. (2012) |
| GS1-10 | 1 | KC796762 | Gaisi, Qinghai, China | This study |
| K-Q4 | 1 | JN685462 | Q block, Kalamay, China | Zhao et al. (2012) |
| D2-18 | 1 | KC796765 | N2, Daqing, China | This study |
| D5-13 | 1 | KC796774 | N2, Daqing, China | This study |
| GD2-9 | 1 | KC796756 | Gudao, Shengli, China | This study |
| D5-22 | 1 | KF131788 | N2, Daqing, China | This study |
| D004023B01 | 1 | EU721796 | Schrader bluff, Alaska, USA | Pham et al. (2009) |
| V4P-10 | 1 | JQ519708 | V4, Henan, China | Zhang et al. (2012) |
| D2-15 | 1 | KC796764 | N2 block, Daqing, China | This study |
| Ling 12-5-1 | 1 | KC796759 | Qiuling, Tuha, China | This study |
| V4P-17 | 1 | JQ519715 | V4 block, Henan, China | Zhang et al. (2012) |
| D4-8 | 1 | KC796771 | N2 block, Daqing, China | This study |
| Kong 1002-1 | 2 | KC796782 | Kongdian, Dagang, China | This study |
| SOB-31 | 2 | AB126364 | Sagara, Japan | Nunoura et al. (2006) |
| N6I-18 | 2 | JQ519744 | N6 block, Xinjiang, China | Zhang et al. (2012) |
| D004023G12 | 3 | EU721804 | Schrader bluff, Alaska, USA | Pham et al. (2009) |
| V4I-18 | 3 | JQ519784 | V4 block, Henan, China | Zhang et al. (2012) |
| Kong1023-3 | 18 | KC796783 | Kongdian, Dagang, China | This study |
| Zhuang 6-2 | A | KC796778 | Yangerzhuang, Dagang, China | This study |
| YC12-1 | A | KC796780 | Yangcong, Dagang, China | This study |
| GD2-2 | A | HM030754 | Gudao, Shengli, China | This study |
| YC27-1 | B | KC796781 | Yangcong, Dagang, China | This study |
| M17-10-B08 | C | JQ088384 | Mengguiling, Shengli, China | Tang et al. ( 2012) |
| C-7 | C | FJ900832 | Gudao, shengli, China | Ren et al. (2012) |
| C-59 | C | FJ900877 | Gudao, shengli, China | Ren et al. (2012) |
| C-64 | C | FJ900882 | Gudao, shengli, China | Ren et al. (2012) |
| B18-44-B04 | D | JQ88445 | Baologe, Shengli, China | Tang et al. ( 2012) |
| B18-44-B03 | D | JQ88449 | Baologe, Shengli, China | Tang et al. ( 2012) |
| D2-21 | D | KC796777 | N2 block, Dagang, China | This study |
| D2-2 | D | KC796760 | N2 block, Dagang, China | This study |
| D2-13 | D | KC796763 | N2 block, Dagang, China | This study |
| GD2-3 | D | KC796766 | Gudao, Shengli, China | This study |
| D2-24 | D | KC796767 | N2 block, Dagang, China | This study |
| D4-3 | D | KC796769 | N2 block, Dagang, China | This study |
| D5-5 | D | KC796772 | N2 block, Dagang, China | This study |
| C-17 | D | FJ900833 | Gudao, Shengli, China | Ren et al. (2012) |
| NK-B25 | D | JN685507 | M block, Huabei, China | Zhao et al. (2012) |
| D4-4 | D | KC796770 | N2 block, Shengli, China | Ren et al. (2012) |
| C-1 | D | FJ900847 | Gudao, Shengli, China | Ren et al. (2012) |
| D4-2 | D | KC796768 | N2 block, Daqing, China | This study |
| C-34 | E | FJ900855 | Gudao, Shengli, China | Ren et al. (2012) |
| NK-M25 | F | JN685487 | M block, Huabei, China | Zhao et al. (2012) |
| K2I-30 | G | AY770970 | Kongdian, Dagang, China | Zhang et al. (2012) |
| Zhuang 7-7 | G | KC796779 | Kongdian, Dagang, China | This study |
| OtuB52 | H | HQ395164 | Bokor, Malaysian | Li et al. (2012) |
| OtuB51 | H | HQ395163 | Bokor, Malaysian | Li et al. (2012) |
| ***P. stutzeri* Isolated from samples in oil reservoirs** | | | | |
| DG2-DNN-2 | 1 | KC796792 | Kongdian, Dagang, China | This study |
| DG1-DNN-2 | 1 | KC796789 | Kongdian, Dagang, China | This study |
| DG1-DAN-3 | 1 | KC796790 | Kongdian, Dagang, China | This study |
| DG1-DNN-2 | 1 | KC796785 | Kongdian, Dagang, China | This study |
| DG1-DNN-1 | 1 | KC796784 | Kongdian, Dagang, China | This study |
| DG2-DAN-1 | 1 | KC796791 | Kongdian, Dagang, China | This study |
| DG2-DAN-3 | D | KC796786 | Kongdian, Dagang, China | This study |
| DQ-DAN-2 | D | KC796787 | N2, Daqing, China | This study |
| DG2-DAN-1 | D | KC796793 | Kongdian, Dagang, China | This study |
| DQ-DAN-1 | D | KC796794 | N2, Daqing, China | This study |
| DG1-DAN-4 | G | KC796788 | Kongdian, Dagang, China | This study |
